# Geography and public health: analysis of the epidemiological dynamics of meningitis in Brazil, between 2010 and 2019

**DOI:** 10.1590/1980-549720240031

**Published:** 2024-06-14

**Authors:** Luis Roberto da Silva, Laís Eduarda Silva de Arruda, Isabel de Jesus Brandão Barreto, João Victor Rodrigues de Aragão, Maria Luiza Ferreira Imburana da Silva, Guilherme Lira, Camila Maria Barros Teixeira, Emília Carolle Azevedo de Oliveira

**Affiliations:** I School of Medicine of Universidade de São Paulo, Postgraduate Program in Public Health – São Paulo (SP), Brazil.; II Fundação Oswaldo Cruz, Instituto Aggeu Magalhães, Multiprofessional Residency Program in Public Health – Recife (PE), Brazil.; III Universidade Federal de Pernambuco, Academic Center of Vitória, Multiprofessional Residency Program for Internalization of Health Care – Vitória de Santo Antão (PE), Brazil.; IV Centro Universitário Uninovafapi – Teresina (PI), Brazil.; V Recife Health Secretariat, Multiprofessional Residency Program in Health Surveillance – Recife (PE), Brazil.; VI Universidade Federal de Pernambuco, Technology and Geosciences Center – Recife (PE), Brazil.; VII Fundação Oswaldo Cruz, Instituto Aggeu Magalhães – Recife (PE), Brazil.

**Keywords:** Meningitis, Vaccine-preventable diseases, Public health, Epidemiologic factors, Time series studies, Spatial analysis

## Abstract

**Objective:**

To analyze the spatiotemporal epidemiological dynamics of meningitis in Brazil, between 2010 and 2019.

**Methods:**

Descriptive ecological study with cases and deaths due to meningitis in Brazil (2010-2019) in the National Notifiable Diseases Information System (*Sistema de Informações de Agravos de Notificação* – SINAN). The following analyses were performed: (I) frequency analyses of cases and deaths, prevalence rates, mortality, lethality, Fisher's exact test, and chi-square test; (II) Prais-Winstein regression; and (III) Global, Local Moran's index, and Kernel density.

**Results:**

182,126 cases of meningitis were reported in Brazil, of which 16,866 (9.26%) resulted in death, with prevalence rates of 9.03/100,000 inhabitants, mortality of 0.84/100,000 inhabitants, and lethality of 9.26%. There was a noted trend of decreasing prevalence rates (−9.5%, 95% confidence interval — 95%CI −13.92; −4.96, p<0.01) and mortality (−11.74%, 95%CI −13.92; −9.48, p<0.01), while lethality remained stable (−2.08%, 95%CI −4.9; 0.8; p<0.1941). The majority of cases were viral meningitis (45.7%), among 1-9 years old (32.2%), while the highest proportion of deaths was due to bacterial meningitis (68%), among 40-59 years old (26.3%). In the Moran and Kernel maps of prevalence and mortality rates, municipalities in the South, Southeast, and the capital of Pernambuco in the Northeast stood out with high rates; as for lethality, the North, Northeast, and Southeast coastal areas were highlighted.

**Conclusion:**

A decrease in meningitis cases and deaths was found in this study; however, the lethality rate was higher in areas with lower prevalence, emphasizing the need to enhance actions for identifying, monitoring, and providing health care for cases, as well as expanding vaccination coverage.

## INTRODUCTION

Meningitis is a condition marked by the inflammation of the meninges and the subarachnoid space, which are protective layers surrounding the central nervous system. These layers consist of three membranes — dura mater, arachnoid mater, and pia mater. Within these layers lies the cerebrospinal fluid (CSF), a vital biological fluid that acts as a cushion for the nervous system^1–3^.

The etiology of meningitis can be linked to various infectious agents, including viruses, bacteria, and other pathogens. However, non-infectious factors such as certain medications and other medical conditions can also trigger this inflammatory process^3,4^.

Viral meningitis is the most common type in the population, as it is caused by a larger variety of pathogens compared to bacterial meningitis. This prevalence is often associated with the socioeconomic vulnerability of the population^5^. However, bacterial meningitis is considered the most severe and potentially lethal form due to its severity compared to the viral type^5,6^.

Meningitis remains a significant public health concern despite being preventable through vaccination. Globally, it is estimated that there are 1.2 million cases of meningitis annually, resulting in approximately 135,000 deaths. Without prompt treatment, its lethality can reach as high as 50%^7^. In Brazil, suspected or confirmed cases of meningitis are subject to immediate mandatory notification, requiring reporting to the State and Municipal Health Secretariats within 24 hours^8^.

Reports of meningitis cases in Brazil exhibit regional disparities, with a higher concentration observed in the South, Southeast, and Northeast regions. This pattern is evident in studies focusing on bacterial meningitis cases as well as those analyzing all types of the disease^9,10^.

Hence, in 2021, the World Health Organization (WHO) initiated a global roadmap with the goal of managing meningitis and decreasing fatalities from the illness by 2030. This initiative seeks to eradicate outbreaks of bacterial meningitis, decrease instances of vaccine-preventable bacterial meningitis, and enhance the quality of life for those impacted by the disease^11^.

Presently, vaccines stand as one of the primary methods to prevent meningitis. In Brazil, these vaccines are integrated into the national immunization schedule of the National Immunization Program (*Programa Nacional de Imunização* – PNI) and are provided free of charge through the Brazilian Unified Health System (*Sistema Único de Saúde* – SUS). Included among these vaccines are the 10-valent pneumococcal (PCV 10), meningococcal C (Conjugated), 23-valent pneumococcal (PPV 23), pentavalent and meningococcal ACWY (Conjugated). Studies demonstrate that vaccination has yielded a beneficial impact in reducing both cases and fatalities from the disease in recent years^13^.

Clinical manifestations of meningitis vary based on factors such as age, duration of illness, and causative agent. Key signs and symptoms include fever, hypothermia, diarrhea, lethargy, neck stiffness (meningeal irritation), lower back pain, photophobia, dyspnea, and the emergence of petechiae and maculopapular rashes^14^. Laboratory confirmation can be achieved through polymerase chain reaction (PCR) tests, latex agglutination, and culture, with the latter considered the gold standard for diagnosing bacterial meningitis^8^.

Given this context, conducting this research is justified due to the significance of understanding the epidemiological dynamics of meningitis in Brazil and contributing to the spatial and temporal comprehension of the disease and its features. Employing geographic, socioeconomic, and health data enables the characterization of epidemiological scenarios relevant to public and collective health^15,16^.

The present study aimed to address the following question: given the status of meningitis as a public health concern, what is the epidemiological landscape of meningitis in Brazil in recent years? Thus, the objective was to analyze the spatiotemporal epidemiological dynamics of meningitis in Brazil between 2010 and 2019.

## METHODS

A descriptive and ecological study was conducted, employing a spatiotemporal approach, to analyze cases and deaths attributed to various types of meningitis (bacterial, viral, and caused by other agents), in Brazil, from 2010 to 2019. The study utilized data sourced from the National Notifiable Diseases Information System (*Sistema de Informação de Agravos de Notificação* – SINAN), which was established in 1993 and has undergone continuous enhancements. SINAN is responsible for reporting diseases listed in the National Compulsory Disease Notification List^17^.

Data collection was conducted using the TabNet platform of the SUS IT Department (DataSUS), specifically under the "Epidemiological and Morbidity" tab. The option "Notifiable Diseases and Diseases — 2007 onwards (SINAN)" was selected, followed by "Meningitis." Subsequently, the data tabulation process was initiated^18^. Population data were obtained from population estimates provided by the Brazilian Institute of Geography and Statistics (*Instituto Brasileiro de Geografia e Estatística* – IBGE)^19^.

As meningitis is a notifiable disease, cases must be reported within 24 hours to the Municipal and State Health Secretariats by healthcare professionals or individuals responsible for public and private health services providing patient care. Additionally, any citizen can report a suspected case. Notification is carried out using the "Meningitis Investigation Form" and registered on Sinan^20^.

Therefore, the study area encompassed Brazil, a country located in South America, with a territorial extension of 8,510,345.538 km^2^ and an estimated population of 210,147,125 inhabitants for the year 2019. Brazil is administratively divided into 5,570 municipalities, 27states, and five geographic regions (North, Northeast, Southeast, South, and Central-West)^19^.

To characterize the general epidemiological scenario of meningitis in Brazil, absolute and relative frequencies of the selected variables were calculated. These variables include gender, age range, race/color, confirmation criteria, etiology, type of meningitis (grouped by the authors based on etiology), case evolution, pregnant woman status, year of symptom onset, geographic region of residence, state of residence, and municipality of residence^18^ (the categories of these variables are included in the supplementary file). Additionally, prevalence rates (per 100,000 inhabitants), mortality coefficients (per 100,000 inhabitants), and lethality rates (per 100%) were computed.

To calculate the prevalence rate and mortality coefficient, the population projection of the respective year was used as the denominator. The total rates were calculated using the sum of the population projections for the ten years analyzed as the denominator.

Fisher's exact test and the chi-square test were utilized to determine whether there are differences between the averages of cases and deaths, with a significance level of 5% (p<0.05). The analyses were conducted using Microsoft Office software Excel 2016^®^ and R version 4.2.0.

To analyze the temporal evolution, the Prais-Winsten generalized linear regression method was employed. In this method, the prevalence, mortality, and lethality rates of meningitis were considered as dependent variables (Y), while the years studied were treated as the independent variable (X), with a 95% confidence interval. This approach allows inference regarding whether the temporal trend is increasing (positive), decreasing (negative), or stationary (when there is no significant difference between the obtained value and zero). For this analysis, R version 4.2.0 was used, along with the *prais* package^21,22^.

In the spatial analysis, global and local Moran indices and Kernel density were calculated. The global Moran index was utilized to ascertain the presence of spatial autocorrelation in the distribution of meningitis prevalence, mortality, and lethality rates. This method is predicated on the null hypothesis (*H*_0_), which posits that spatial factors do not influence the distribution of the disease, event, or condition. Additionally, values obtained in this analysis range between −1 and 1, with values closer to 1 indicating a stronger presence of spatial autocorrelation, while values close to zero suggest that *H*_0_ is true^23,24^. To validate the findings, a random permutation test with 9,999 permutations and a 95% confidence interval was conducted^24^.

The local Moran index enables the visualization of clusters of municipalities with similar behavior patterns, based on the statistical significance criterion^24^. Meanwhile, the Kernel density tool was employed to pinpoint areas of rate concentration in geographic space. This method utilizes statistical analysis by interpolating point data per unit area to estimate density curves and convert them into data on a continuous surface^25^.

The current study did not require approval from the Research Ethics Committee, as it exclusively utilized secondary data available in the public domain. According to Resolution no. 510/16 of the National Health Council (*Conselho Nacional de Saúde* – CNS), research of this nature is exempt from the requirement for CEP approval^26^.

## RESULTS

Between 2010 and 2019, a total of 182,126 cases of meningitis were reported in Brazil. Among these cases, 16,866 (9.26%) resulted in death. The average prevalence rate during this period was 9.03 cases per 100,000 inhabitants, with an average mortality coefficient of 0.84 deaths per 100,000 inhabitants. The average lethality rate was 9.26%.

Regarding the epidemiological characterization of the cases, it was observed that the majority occurred in male individuals (59.12%). The highest proportion was in the age group from one to nine years (32.18%), and the majority were of white race/color (45.17%). The largest portion of cases had viral etiology (45.69%), confirmed through chemocytological examination (60.99%). Additionally, 79.37% of cases progressed to cure (Table 1).

**Table 1:** Epidemiological characterization of cases and deaths from meningitis in Brazil, between 2010 and 2019.

Characteristics		Cases		Deaths		p-value
		n	%	n	%	
		182,126	100	16,866	100	
Gender						
	Male	107,671	59.12	9,905	58.73	0.5677*
	Female	74,429	40.87	6,959	41.26	
	Ignored	26	0.01	2	0.01	
Age range (years)						
	<1	27,117	14.89	1,912	11.34	<0.01†
	1 to 9	58,608	32.18	2,214	13.13	
	10 to 19	23,172	12.72	1,652	9.79	
	20 to 39	35,309	19.39	3,860	22.89	
	40 to 59	25,412	13.95	4,442	26.34	
	60 and more	12,340	6.78	2,769	16.42	
	Ignored/blank	168	0.09	17	0.10	
Race/color						
	White	82,274	45.17	6,605	39.16	<0.01†
	Black	7,447	4.09	1,150	6.82	
	Brown	53,862	29.57	6,089	36.10	
	Yellow	895	0.49	94	0.56	
	Indigenous	531	0.29	92	0.55	
	Ignored/blank	37,117	20.38	2,836	16.81	
Confirmation criteria						
	Culture	23,334	12.81	4,917	29.15	<0.01†
	IEC	492	0.27	130	0.77	
	Latex agglutination	6,597	3.62	1,469	8.71	
	Clinical	17,655	9.69	3,112	18.45	
	Bacterioscopy	4,554	2.50	649	3.85	
	Chemocytological	111,076	60.99	3,623	21.48	
	Clinical-epidemiological	2,957	1.62	264	1.57	
	Viral isolation	416	0.23	11	0.07	
	PCR — viral	10,723	5.89	1,720	10.20	
	Other technique	3,878	2.13	877	5.20	
	Ignored/blank	444	0.24	94	0.56	
		**n**	**%**	**n**	**%**	
		**182,055** ‡	**100%**	**16,861** ‡	**100%**	
Etiology						
	Meningococcemia	5,202	2.86	1,903	11.28	<0.01†
	Meningococcal meningitis	7,194	3.95	889	5.27	
	Meningococcal meningitis + Meningococcemia	5,426	2.98	1,031	6.11	
	Tuberculous meningitis	3,665	2.01	661	3.92	
	Bacterial meningitis	28,516	15.66	3,735	22.15	
	Unspecified meningitis	28,895	15.87	2,824	16.74	
	Viral meningitis	83,218	45.69	1,057	6.27	
	Meningitis due to other etiologies	7,541	4.14	1,461	8.66	
	Hemophilus meningitis	1,308	0.72	211	1.25	
	S. *pneumoniae* meningitis	10,481	5.75	3,035	17.99	
	Ignored/blank	609	0.33	54	0.32	
		**n**	**%**			
		**182,126**	**100%**			
Evolution						
	High	144,554	79.37	-	-	
	Death from meningitis	16,866	9.26	-	-	
	Death from another cause	6,853	3.76	-	-	
	Ignored/blank	13,853	7.61	-	-	-
		**n**	**%**	**n**	**%**	
		**182,126**	**100%**	**16,866**	**100%**	
Pregnant						
	1^st^ trimester	185	0.10	24	0.14	<0.01†
	2^nd^ trimester	312	0.17	32	0.19	
	3^rd^ trimester	175	0.10	26	0.15	
	Gestational age unknown	90	0.05	8	0.05	
	No	25,744	14.14	3,327	19.73	
	Not applicable	150,527	82.65	12,771	75.72	
	Ignored/blank	5,093	2.80	678	4.02	

* Fisher's exact test;

† χ^2^ test;

‡ The online database showed a divergence in the total number of cases and deaths, when the data for the "etiology" variable were tabulated.

Source: Ministry of Health/Health Surveillance Secretariat — Sinan Net. Prepared by the authors, 2022.

Regarding deaths (n=16,866), the majority occurred in males (58.73%), with the highest proportion in the age range of 40 to 59 years (26.34%). Most of the deceased were of white race/color (39.16%). The main cause of these deaths was bacterial meningitis (22.15%), and the most commonly used confirmation method was culture (29.15%) (Table 1).

**Figure 1:**
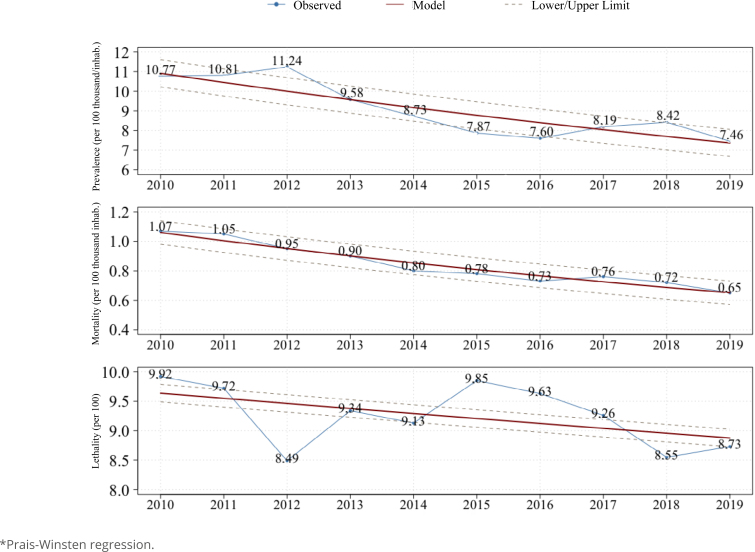
Time series* of meningitis prevalence, mortality, and lethality rates in Brazil, between 2010 and 2019.

Source: Ministry of Health/Health Surveillance Secretariat — Sinan Net. Prepared by the authors, 2023.

In the temporal trend analysis, a decrease in the annual percentage variation in the prevalence rate of −9.5% (95% CI −13.92; −4.96, p<0.01) was observed over the period studied. Similarly, the mortality rate showed a significant decrease of −11.74% (95% CI −13.92; −9.48, p<0.01). Although the lethality rate demonstrated a stationary trend, there was a decrease of −2.08% (95% CI −4.9; 0.8; p<0.1941) over the study period (Figure 1).

Furthermore, when observing the stratification by type of meningitis (Table 2), a stationary trend in the prevalence of viral meningitis and a decreasing trend in bacterial meningitis and other etiological agents were noted. For mortality, a stationary trend was also observed for viral meningitis, whereas a decreasing trend was observed for bacterial meningitis and other types. Regarding lethality, all types of meningitis showed a stationary trend.

**Table 2:** Temporal evolution of prevalence, mortality and lethality rates of types of meningitis in Brazil, between 2010 and 2019.

Rates		Rates		APC*	95%CI*	p-value	Trend
		2010	2019				
Prevalence							
	Viral meningitis	4.36	3.68	-5.5	-5.26; −5.66	0.077	Stationary
	Bacterial meningitis	4.11	2.27	-14.2	-14.03; −14.35	< 0.01	Decreasing
	Other types of meningitis	2.25	1.50	-10.5	-10.31; −10.77	< 0.01	Decreasing
	Ignored/blank	0.04	0.01	-31.2	-29.42; −32.9	0.126	Stationary
	Total	10.76	7.46	-9.5	-9.36; −9.72	< 0.01	Decreasing
Mortality							
	Viral meningitis	0.06	0.05	-1.1	-0.78; −1.42	0.654	Stationary
	Bacterial meningitis	0.76	0.42	-14.4	-14.18; −14.52	< 0.01	Decreasing
	Other types of meningitis	0.24	0.18	-5.9	-5.82; −6.02	< 0.01	Decreasing
	Ignored/blank	0.01	0.01	–	–	–	–
	Total	1.07	0.65	-11.8	-11.65; −11.87	< 0.01	Decreasing
Lethality							
	Viral meningitis	1.26	1.38	8.0	8.41; 7.49	0.122	Stationary
	Bacterial meningitis	18.56	18.38	-0.3	-0.28; −0.4	0.507	Stationary
	Other types of meningitis	10.89	11.93	3.6	3.87; 3.37	0.328	Stationary
	Ignored/blank	13.92	35.48	27.3	29.71; 24.91	0.348	Stationary
	Total	9.93	8.73	-2.1	-1.97; −2.23	0.194	Stationary

* Prais-Winsten regression.

Source: Ministry of Health/Health Surveillance Secretariat — Sinan Net. Prepared by the authors, 2023.

Regarding the analysis of the prevalence of meningitis, according to geographic region, it was observed that the South Region had a rate of 12.48 cases per 100 thousand inhabitants, followed by the Southeast Region (11.65 cases per 100 thousand inhabitants), Northeast Region (5.59 cases per 100 thousand inhabitants), Central-West Region (5.54 cases per 100 thousand inhabitants), and North Region (4.54 cases per 100 thousand inhabitants).

For the mortality coefficient, the Southeast Region stood out with a rate of 1.08 deaths per 100 thousand inhabitants, followed by the South Region (0.89 deaths per 100 thousand inhabitants), Central-West Region (0.67 deaths per 100 thousand inhabitants), North Region (0.65 deaths per 100 thousand inhabitants), and Northeast Region (0.54 deaths per 100 thousand inhabitants). In terms of lethality, the North Region had the highest rate at 14.21%, followed by the Central-West Region (12.08%), Northeast Region (9.73%), Southeast Region (9.25%), and South Region (7.15%).

**Figure 2:**
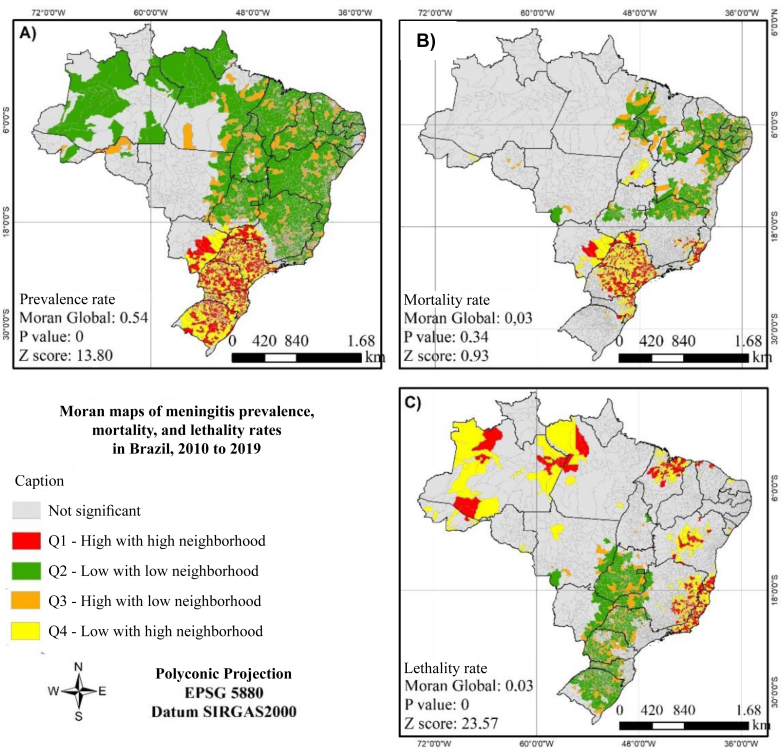
Moran index maps of prevalence (A), mortality (B), and lethality (C) rates of meningitis in Brazil, 2010 and 2019.

Source: Ministry of Health/Health Surveillance Secretariat — Sinan Net. Prepared by the authors, 2022.

The presence of spatial autocorrelation in the distribution of the meningitis prevalence rate was confirmed by the Global Moran index (I=0.54; p-value=0; z-score=13.80). In the local Moran analysis, municipalities in the states of São Paulo, Minas Gerais, Rio de Janeiro, Espírito Santo, Mato Grosso do Sul, Paraná, Santa Catarina, Rio Grande do Sul, and Pernambuco stood out, showing clusters of municipalities with high rates surrounded by neighboring municipalities with similarly high rates (Figure 2A).

Regarding the mortality coefficient, weak spatial autocorrelation was identified (I=0.03; p-value=0.34; z-score=0.93). According to the local Moran map, similar patterns were observed compared to the prevalence rate map, with municipalities in the states of São Paulo, Minas Gerais, Rio de Janeiro, Espírito Santo, Mato Grosso do Sul, Paraná, and Santa Catarina standing out (Figure 2B).

In the lethality map, the presence of fragile spatial autocorrelation was also confirmed (I=0.03; p-value=0; z-score=23.57). However, a change in the highlighted areas of the map was observed. Some areas that did not stand out in the previously described maps emerged in the lethality map, such as municipalities in the states of Amazonas, Pará, Maranhão, Bahia, Minas Gerais, Rio de Janeiro, and Espírito Santo (Figure 2C).

**Figure 3:**
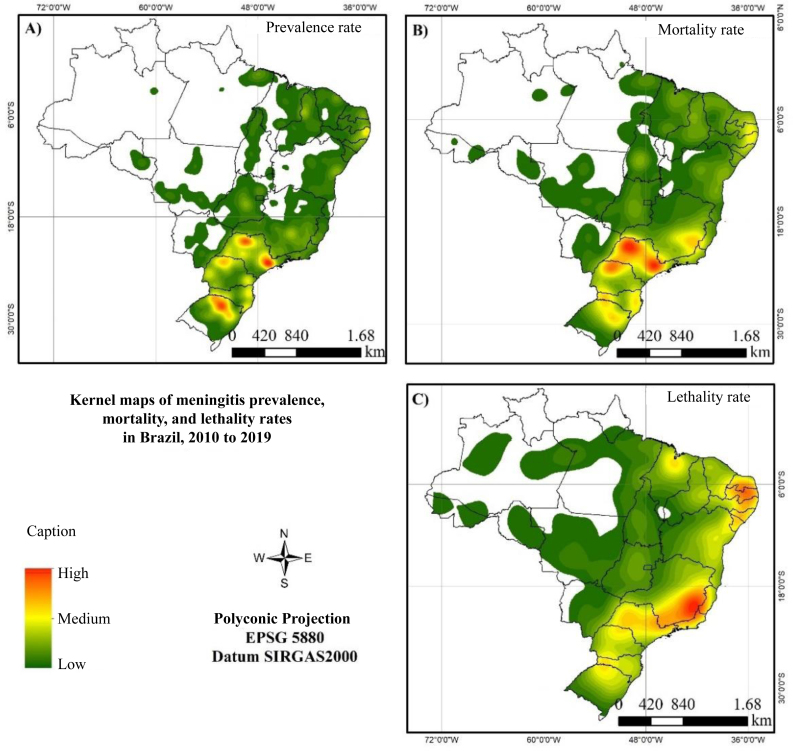
Kernel density maps of prevalence (A), mortality (B), and lethality (C) rates of meningitis in Brazil, 2010 and 2019.

Source: Ministry of Health/Health Surveillance Secretariat — Sinan Net. Prepared by the authors, 2022.

Kernel density analysis of the prevalence rate revealed that certain municipalities in the states of São Paulo, Paraná, Santa Catarina, and Rio Grande do Sul exhibit high and medium density areas (Figure 3A). These areas were also common to the mortality coefficient map, showing high and medium concentrations of deaths, along with the states of Pernambuco, Minas Gerais, and Rio de Janeiro (Figure 3B). Concerning lethality, the highest density areas stood out in the states of Minas Gerais and Paraíba, while municipalities in the states of São Paulo, Minas Gerais, Paraná, Santa Catarina, Rio Grande do Sul, Maranhão, and some municipalities along the northeastern coast exhibited areas of medium concentration (Figure 3C).

## DISCUSSION

Based on the analyses conducted in this study, it was observed that there is a trend toward a decrease in the prevalence and mortality rates of meningitis in Brazil. However, lethality rates remain stationary, posing a challenge for health authorities. Additionally, there was a higher prevalence of cases attributed to viral meningitis, while bacterial meningitis accounted for greater mortality. Moreover, a greater concentration of cases was found in the states of the South and Southeast Regions.

As supported by the scientific literature, the study also identified a higher concentration of meningitis cases among children. This observation may be associated with factors such as the development of the immune system, incomplete vaccination schedules, and the close proximity of children in educational settings, which can facilitate the transmission of infectious agent(s)^3,6^.

In relation to the criterion of race/skin color, existing literature indicates a predominance of cases among individuals of white ethnicity, consistent with the findings of this study. However, it is important to acknowledge that this observation may vary across different studies due to factors such as the demographic composition of specific regions, the ethnic diversity of the Brazilian population, and issues related to data reporting. It is noteworthy that a considerable proportion of cases (more than 20%) and deaths (16%) had this field left blank in the Sinan notification records, which may introduce some uncertainty into the analysis^8,27^.

The research also identified that the majority of recurrent meningitis cases in the population are viral in nature, potentially attributed to the virus's ability to spread easily in the environment. Existing literature supports this observation, noting that viral meningitis is more prevalent and typically presents with milder symptoms. However, bacterial meningitis remains a significant concern due to its considerable morbidity and mortality rates, particularly among children in regions characterized by higher economic and social vulnerability^2,3,23,28^.

Advancements in diagnostic techniques and the continuous training of healthcare professionals are essential strategies for the prompt detection of meningitis cases in the population. Early detection enables timely initiation of treatment, increasing the likelihood of a complete recovery without long-term complications and reducing mortality rates associated with the disease^27,29^.

According to the epidemiological bulletin from the Ministry of Health^8^, there has been a notable decline in the incidence of pneumococcal meningitis cases in Brazil since the introduction of PCV-10 vaccination in 2010. The incidence dropped from 2.5 cases per 100 thousand inhabitants in 2007 to 1 case per 100 thousand inhabitants in 2015, with rates remaining relatively stable in subsequent years. This underscores the significance of population vaccination as the primary strategy for mitigating and controlling meningitis cases across Brazil.

The spatial analysis revealed a clustering of meningitis cases and deaths in states located in the South (Paraná, Santa Catarina, and Rio Grande do Sul), Southeast (São Paulo, Minas Gerais, Rio de Janeiro, Espírito Santo), and Central-West (Mato Grosso do Sul) regions. This concentration could be attributed to several factors, including the advancement of health surveillance methods in these regions for early case detection and/or higher population density, particularly notable in the Southeast. These conditions may facilitate the transmission of infectious agents within the population^3,27,30^.

The contrast between the lethality map and the prevalence/mortality maps is noteworthy. Areas with lower prevalence or mortality rates, particularly in the North and Northeast Regions, exhibited higher lethality rates. This observation might be linked to challenges within the health system to promptly identify cases, thus preventing adverse outcomes such as death. Additionally, factors such as low vaccination coverage and socioeconomic and regional disparities could contribute to these higher lethality rates. These regions are historically marked by socioeconomic inequality and health disparities, which may exacerbate the impact of meningitis^13,31^.

The study's limitations include the potential for biases inherent in the notification and updating of cases and deaths on Sinan, which may not accurately reflect the epidemiological landscape of meningitis in Brazil. Additionally, the presence of fields not filled in or ignored in a percentage of notifications poses challenges in understanding the true extent of meningitis in the country.

To mitigate these limitations, inconsistencies and incompleteness in the database were rigorously analyzed using methodological procedures, with support from the existing scientific literature on the subject. This approach aimed to ensure a robust interpretation of the findings.

In summary, there was a trend toward a decline in both the prevalence and mortality rates of meningitis. However, the persistent lethality rates warrant careful attention, especially in regions where lower prevalence rates coincide with higher lethality rates. This concern arises from the fact that lethality reflects the percentage of individuals who succumb to meningitis after falling ill.

Therefore, it is crucial to intensify efforts to enhance public health services and interventions across the country to promptly detect cases of meningitis, thereby mitigating mortality rates associated with the disease. Moreover, expanding vaccination coverage against vaccine-preventable diseases among the population is imperative. Finally, it is recommended to conduct further research on the epidemiology of meningitis in Brazil, focusing on assessing the impact of vaccination on meningitis cases and deaths, as well as comparing the epidemiological dynamics of different types of meningitis.

## Supplementary Material


