# Supplementary material for: Geography and public health: analysis of the epidemiological dynamics of meningitis in Brazil, between 2010 and 2019
Source: Rev Bras Epidemiol. 2024 Jun 14;27:e240031. doi: 10.1590/1980-549720240031 (PMC11182441; doi:10.1590/1980-549720240031)
Supplement: Supplementary file 1 [file 1980-5497-rbepid-27-e240031-Suppl01.docx]

Quadro 1 - Variáveis escolhidas para o estudo e as suas categorias.

| **Variável** | **Categorias** |
| --- | --- |
| **Sexo** | Masculino; feminino e ignorado. |
| **Faixa etária** | < 1 ano (menores de um ano de idade); 1 a 9 anos; 10 a 19 anos; 20 a 39 anos; 40 a 59 anos; 60 anos e mais e ignorados/em branco. |
| **Raça/cor** | Branca; preta; parda; amarela; indígena e ignorado/em branco. |
| **Critério de confirmação** | Cultura; Contraimunoeletroforese Cruzada (CIE); Aglutinação do látex; clínico; bacterioscopia; quimiocitológico; clínico-epidemiológico; isolamento viral; Reação em Cadeia da Polimerase-viral (PCR-viral); outra técnica e ignorado/em branco. |
| **Etiologia** | Meningococcemia; Meningite meningocócica; Meningite; Meningocócica + Meningococcemia; Meningite tuberculosa; Meningite bacteriana; Meningite não especificada; Meningite viral; Meningite por outras etiologias; Meningite por hemófilo; Meningite por S. *pneumoniae* e ignorado/em branco. |
| **Tipo de meningite^*^** | Meningite viral; Meningite bacteriana; outros tipos de meningite; ignorado/em branco. |
| **Evolução do caso** | Alta; óbito por meningite; óbito por outra causa e ignorado/em branco. |
| **Gestante** | 1º trimestre; 2º trimestre; 3º trimestre; idade gestacional ignorada; não; não se aplica; e ignorado/em branco. |
| **Ano de início dos sintomas** | 2010, 2011, 2012, 2013, 2014, 2015, 2016, 2017, 2018, 2019. |
| **Região geográfica de residência** | Norte, Nordeste, Sudeste, Sul e Centro-Oeste. |
| **Unidade da Federação de Residência (UF)** | Acre; Amapá, Amazonas, Pará, Rondônia, Roraima, Tocantins, Alagoas, Bahia, Ceará, Maranhão, Paraíba, Pernambuco, Piauí, Rio Grande do Norte, Sergipe, Espírito Santo, Minas Gerais, Rio de Janeiro, São Paulo, Paraná, Rio Grande do Sul, Santa Catarina, Distrito Federal, Goiás, Mato Grosso, Mato Grosso do Sul. |
| **Município de residência** | Todos os municípios do Brasil. |

Fonte: Ministério da Saúde/Secretaria de Vigilância em Saúde - Sinan Net. Elaborado pelos autores, 2022.

*Agrupado pelos autores a partir da etiologia da meningite.
